# Expression patterns of key Sonic Hedgehog signaling pathway components in the developing and adult mouse midbrain and in the MN9D cell line

**DOI:** 10.1007/s00441-017-2664-2

**Published:** 2017-08-11

**Authors:** Melanie Feuerstein, Enaam Chleilat, Shokoufeh Khakipoor, Konstantinos Michailidis, Christian Ophoven, Eleni Roussa

**Affiliations:** 1 Institute of Anatomy and Cell Biology, Department of Molecular Embryology, Faculty of Medicine, University of Freiburg, Albertstrasse 17, D-79104 Freiburg, Germany; 2 Neuroanatomy, Faculty of Medicine, University of Freiburg, Albertstrasse 17, D-79104 Freiburg, Germany

**Keywords:** Gas1, Development, Cell fate, Dopaminergic neurons, Gli

## Abstract

The temporal dynamic expression of Sonic Hedgehog (SHH) and signaling during early midbrain dopaminergic (mDA) neuron development is one of the key players in establishing mDA progenitor diversity. However, whether SHH signaling is also required during later developmental stages and in mature mDA neurons is less understood. We study the expression of SHH receptors *Ptch1* and *Gas1* (growth arrest-specific 1) and of the transcription factors *Gli1*, *Gli2* and *Gli3* in mouse midbrain during embryonic development [embryonic day (E) 12.5 onwards)], in newborn and adult mice using in situ hybridization and immunohistochemistry. Moreover, we examine the expression and regulation of dopaminergic neuronal progenitor markers, midbrain dopaminergic neuronal markers and markers of the SHH signaling pathway in undifferentiated and butyric acid-treated (differentiated) MN9D cells in the presence or absence of exogenous SHH in vitro by RT-PCR, immunoblotting and immunocytochemistry. *Gli1* was expressed in the lateral mesencephalic domains, whereas *Gli2* and *Gli3* were expressed dorsolaterally and complemented by ventrolateral expression of *Ptch1*. Co-localization with tyrosine hydroxylase could not be observed. GAS1 was exclusively expressed in the dorsal mesencephalon at E11.5 and co-localized with Ki67. In contrast, MN9D cells expressed all the genes investigated and treatment of the cells with butyric acid significantly upregulated their expression. The results suggest that SHH is only indirectly involved in the differentiation and survival of mDA neurons and that the MN9D cell line is a valuable model for investigating early development but not the differentiation and survival of mDA neurons.

## Introduction

Among the neurotransmitter systems in the brain, the midbrain dopaminergic system is of particular importance because of its relevance for several neuropsychiatric disorders. Ventral midbrain dopaminergic (mDA) neurons play a pivotal role in several brain functions, among them voluntary movements, reward behaviors, cognition and emotions (Björklund and Dunnett 2007; Morales and Margolis 2017; Vogt Weisenhorn et al. 2016). Degeneration of the tyrosine hydroxylase (TH)-producing neurons in the substantia nigra pars compacta (SNC; A9 group) is the hallmark of Parkinson’s disease (PD; Poewe et al. 2017). Since cell replacement therapy has emerged as a promising therapeutic strategy for PD, understanding the structure and function of these neurons is a prerequisite for tissue engineering approaches. The molecular mechanisms underlying induction, differentiation and survival of mesencephalic dopaminergic neurons have been extensively studied. It is well established that induction of mDA neurons from progenitor cells depends on the temporal and spatial expression of patterning molecules, such as SHH and FGF8, which act as extrinsic determinants and of a subsequent activation of a combinatorial code of transcription factors representing the intrinsic determinants (reviewed in Smidt and Burbach 2007). Interestingly, during recent years, a molecular and functional heterogeneity has been experimentally identified, not only between neurons of A9 and A10 (ventral tegmental area) groups but also within subsets of individual mDA neurons within the groups (reviewed in Blaess and Ang 2015; Fu et al. 2016; Hegarty et al. 2013; Morales and Margolis 2017; Vogt Weisenhorn et al. 2016). mDA neuron subgroups may differ considerably with regard to their morphology, localization and projections but also with regard to their function, as reflected by distinct electrophysiological properties, multiplexed neurotransmission (reviewed in Trudeau et al. 2014; Barker et al. 2016; Tritsch et al. 2016) and also differential gene expression within dopaminergic subpopulations. Subsequently, these observations provide a more complete and at the same time more complex composition of this neuronal complex. Importantly, individual subpopulations modulate distinct behaviors (Morales and Margolis 2017).

During CNS development, the patterning molecule Sonic Hedgehog (SHH) contributes to the establishment of the polarity at the dorsoventral axis and regulates numerous developmental processes (Cohen et al. 2013). The SHH signaling pathway regulates the early induction and expansion of progenitors in several brain areas, such as the ventral forebrain, midbrain and midbrain/hindbrain boundary. With regard to the development of mDA neurons, SHH acts together with other extrinsic cues, such as FGF8 and TGF-β and has been identified as a critical molecular player for midbrain progenitors to acquire a dopaminergic cell fate in vitro and in vivo (Farkas et al. 2003; Roussa et al. 2004, 2006). Moreover, the temporal dynamic expression of SHH expression and signaling during mDA neuron development is one of the key determinants in establishing mDA progenitor diversity (Blaess et al. 2011; Joksimovic et al. 2009a). However, whether SHH signaling is also required during later developmental stages is less understood and SHH has increasingly been considered to function as an early patterning molecule for the mDA progenitor domain (floor plate) rather than acting directly on the differentiation and survival of mDA neurons (Mesman et al. 2014, Blaess et al. 2006, 2011; Tang et al. 2013). SHH reveals a gradient expression and the Patched 1 (Ptch1) protein is the ligand-binding component of the hedgehog receptor complex. In the absence of hedgehog binding, Ptch1 is thought to sustain Smoothened (Smo) in an inactive state and to inhibit signaling to downstream target genes (Chen and Struhl 1996). Upon binding of SHH to Ptch1, Ptch1-regulated inhibition of Smo is abolished and regulation of SHH target genes is induced, including the Gli protein family members, Gli1, Gli2 and Gli3 (Ingham and McMahon 2001). In addition to Ptch1, other membrane-associated proteins, such as Cdon (cell adhesion molecule-related/downregulated by oncogenes), Boc (biregional Cdon-binding protein) and Gas1 (growth arrest-specific 1) are identified to function as accessory receptors that are able to modulate SHH signaling activity (Tenzen et al. 2006). Whether Gas1 is expressed in the developing and mature mouse mDA neurons has not been yet elucidated.

The aim of the present study was to extend studies on expression of molecules associated with SHH signaling and analyze expression patterns of SHH receptors (*Ptch1* and *Gas1*) and the downstream SHH targets *Gli1*, *Gli2* and *Gli3*, in the developing mouse midbrain from E12.5 onwards in newborn and adult mice. Moreover, since MN9D cells are commonly used as a model to investigate mDA neurons, we examined the expression and regulation of dopaminergic neuronal progenitor markers (Msx1, Ngn2), midbrain dopaminergic neuronal markers (Nurr1, Pitx3, Dat, Vmat2) and markers of SHH signaling pathway (Ptch1, Gli1, Gli2 and Shh) in undifferentiated and butyric acid-treated (differentiated) MN9D cells in the presence or absence of exogenous SHH in vitro*.*

## Materials and methods

### Antibodies and reagents

Rabbit polyclonal anti-TH (AB152), mouse monoclonal anti-TH (MAB5280) and sheep polyclonal anti-TH (AB1542) were purchased from Merck-Millipore (Darmstadt, Germany), goat polyclonal anti-Gas1 (AF2644) was from R&D Systems (Wiesbaden, Germany), rabbit polyclonal anti-Ptch1 (ab53715; Gao et al. 2014), anti-Gli1 (ab49314; Smirnova et al. 2016), anti-Gli2 (ab167389 for western blots), anti-Gli3 (ab6050 for western blots and ab69838 for immunocytochemistry; Pan et al. 2015), anti-Ki67 (ab16667), mouse monoclonal anti-GAPDH (ab8245) and anti-Nestin (ab11306; Neri et al. 2015) were purchased from Abcam (Cambridge, UK), rabbit polyclonal anti-βIII-tubulin (T2200; Rutherford et al. 2016) was from Sigma (Munich, Germany), rabbit polyclonal anti-Gli2 (18989–1-AP for immunocytochemistry; Pang et al. 2015) was from Proteintech and rabbit polyclonal anti-Nurr1 (sc991; Roussa et al. 2006) was from Santa Cruz and were used as primary antibodies. Donkey anti-rabbit IgG, donkey anti-goat IgG or donkey anti-mouse IgG Alexa 594 or Alexa 488 or Alexa 568 and donkey anti-sheep IgG coupled to biotin were from Dianova (Hamburg, Germany) and were used as secondary antibodies for immunohistochemistry and immunocytochemistry. Goat anti-rabbit (Cell Signaling, Danvers, MA, USA) and goat anti-mouse coupled to horseradish peroxidase (GE Healthcare, Amersham, UK) were used as secondary antibodies for western blot. DAPI-Fluoromount-G was from Biozol (Eching, Germany) and Aquatex from Merck-Millipore (Darmstadt).

### Animals

This study was carried out in strict accordance with the National Health and ethical regulations and care of animals was in accordance with institutional guidelines. The protocol was approved by the Committee on the Ethics of Animal Experiments of the University of Freiburg (Permit Number: X-13/09H). Pregnant C57BL/6 J mice were sacrificed by cervical dislocation and embryos at embryonic day (E)11.5, E12.5, E13.5 or E17.5 were collected in PBS. The day of vaginal plug identification was designated E1. Newborn (P0) and adult (2–3 months old) mice were fixed with 4%PFA/PBS and the brain was quickly excised. Embryos, embryo heads or brains were (post-)fixed in 4%PFA/PBS overnight. Subsequently, fixed embryos or brains were cryoprotected in 15 and 30% sucrose, frozen in liquid nitrogen and cut into 10-μm serial coronal sections.

### In situ hybridization

Non-radioactive in situ hybridization on cryosections and preparation of digoxigenin-labeled probes were carried out as described by Ernsberger et al. (1997). Riboprobes were labeled with a digoxigenin labeling kit (Roche, Mannheim, Germany) and revealed by BCIP/NBT (Roche). *Gli1*, *Gli2*, *Gli3* and *Ptch1* ISH probes were kindly provided by Dr. Alexandra L.Joyner (Howard Huges Medical Institute, Skirball Institute of Biomolecular Medicine, New York, USA; Platt et al. 1997) and the *Neurofilament* ISH probe was provided by Dr. Katrin Huber (Department of Medicine, University of Fribourg, Fribourg, Switzerland; Huber et al. 2002). Subsequently, sections were processed for immunohistochemistry as described below.

### Immunohistochemistry and immunofluorescence on fixed sections

Immunohistochemistry was performed after ISH in fixed tissue sections. Sections were washed with PBS for 10 min. After blocking endogenous peroxidase activity by 30-min treatment with 3% H_2_O_2_ in H_2_O, sections were washed with PBS and incubated with a sheep polyclonal anti-TH antibody diluted at 1:500 in blocking solution [1.5% normal donkey serum (NDS) + 0.2% Triton-X 100/PBS] overnight at 4 °C. Sections were rinsed 3 × 10 min in 0.2% Triton-X 100/PBS and incubated with biotinylated secondary antibody at dilution 1:200 for 2 h at RT, followed by incubation with Vectastain ABC reagent for 45 min. Horseradish peroxidase reaction was visualized by 3-amino-9-ethylcarbazole. Sections were rinsed with Aqua dest. and mounted using Aqua Tex.

For double immunofluorescence, cryosections were washed with PBS, treated with 1% Triton-X 100/PBS for 15 min, blocked with 4% BSA for 1 h at RT and incubated with primary antibodies (either anti-Gas1 1:100 and anti-TH 1:200, or anti-Gas1 1:100 and Ki67 1:100) in blocking solution overnight at 4 °C. After washing with PBS, slides were incubated with donkey anti-goat IgG Alexa Fluor 594 and either donkey anti-mouse IgG Alexa Fluor 488 or donkey anti-rabbit IgG Alexa Fluor 488 as secondary antibodies at dilution 1:400 in 1.5% NDS/PBS for 1 h at RT. Slides were washed with PBS and mounted with Fluoromount-G, containing 4′,6′-diamidino-2-phenylindole dihydrochloride (DAPI), for nuclear staining. Slides were viewed with a Zeiss Axioplan 2 epifluorescence microscope (Göttingen, Germany).

### Cell culture

The MN9D cell line, a hybridoma cell line established by fusing embryonic primary cells from mouse ventral midbrain with cells from the mouse neuroblastoma cell line N18TG2 (Choi et al. 1991), was used for in vitro experiments. Cells were plated on poly-D-lysine-coated wells or coverslips and cultured in DMEM/F-12 1:1, supplemented with 10% FBS and 1% PSN. Cells were passaged when confluent and incubated in a 5% CO_2_ /95% O_2_ atmosphere at 37 °C. Cells were allowed to differentiate by treating with 1 mM butyric acid (BA) for at least 6 days (Dong et al. 2008). Undifferentiated and differentiated MN9D cells were subsequently treated with 1 nM SHH (R&D Systems) for 48 h. Control and SHH-treated cells were either fixed for immunofluorescence, or processed for RNA extraction and RT-PCR, or processed for protein extraction and immunoblotting.

### Immunocytochemistry

Immunocytochemistry on MN9D cells was performed essentially as described earlier (Roussa et al. 2006). Control, BA- and SHH-treated cells were fixed in 4% PFA/PBS for 30 min at RT, washed with PBS, treated with 1%SDS/PBS for 5 min, blocked with 1%BSA/PBS for 15 min and incubated with primary antibodies overnight at 4 °C (anti-Gli1, anti-Gli2, anti-Gli3, anti-Nestin and anti-Ptch1 1:100, anti-βIII-tubulin and anti-Nurr1 1:200 and anti-TH 1:500 in blocking solution). Cells were washed with PBS and incubated with donkey anti-rabbit IgG Alexa Fluor 568 1:400 for 1 h at RT. Cells were washed in PBS, mounted with Flouromount-G containing DAPI and viewed with a Leica SP8 confocal microscope. Control experiments for labeling specificity were performed by omitting the primary antibody.

### Image acquisition and analysis

Images were acquired with a Leica TCS SP8 confocal microscope using a CS2 63 × 1.40 oil objective lens. Immunofluorescence intensity following treatments was determined for each antibody. Within each experiment, confocal microscope settings (laser power, detector gain and amplifier offset) were kept the same for all scans in which protein expression was compared. Z-stacks of five or six optical sections with a step size of 1 μm were taken for at least 4 separate fields of view for each experimental condition. Maximum intensity projections were created from the z-stacks. To quantify protein expression, ImageJ (NIH) was used to measure the average intensity within the soma. Only differentiated cells were included in the quantification. Background subtraction was applied to the images. After quantification, data were normalized to the mean of controls. Representative images in each figure were processed identically.

### RT-PCR

Total RNA was isolated from control undifferentiated and differentiated MN9D cells and from cells treated with 1 nM SHH, reverse transcribed and processed for PCR, as described earlier (Osterberg et al. 2011). The following primers were used: *Gli1* (Genebank accession number NM 010296.2) Forward: 5′- ACTGGGGTGAGTTCCCTTCT-3′ (nt 2500–2519), Reverse: 5′- TGGCAGGGCTCTGACTAACT-3′ (nt 2991–2972). *Gli2* (Genebank accession number NM_001081125.1) Forward: 5′- CCCCCTAGCATCAATGAGAA-3′ (nt 3389–3408), Reverse: 5′- TCTGCACGGATTGTGGATT-3′ (nt 3884–3866). *Ptch1* (Genebank accession number XM 006517163.3): Forward: 5′- AAAGAACTGCGGCAAGTTTTTG-3′ (nt 260–281), Reverse: 5′- CTTCTCCTATCTTCTGACGGGT-3′ (nt 423–402). *Shh* (Genebank accession number NM 009170.3): Forward: 5′- CTGGCCAGATGTTTTCTGGT-3′ (nt 351–370), Reverse: 5′- GATGTCGGGGTTGTAATTGG-3′ (nt 593–574). *Gapdh (*Genebank accession number NM_001289726.1): Forward: *5′-* TGACGTGCCGCCTGGAGAAA -3′ (nt 820–839), Reverse: 5′- AGTGTAGCCCAAGATGCCCTTCAG-3′ (nt 917–894). *Nurr1* (Genebank accession number NM_013613.2): Forward: 5′- TCTGGAGTTAAGAAATCGGAGCTG-3′ (nt 558–535), Reverse: 5′- TGAAGAGAGCGGAGAAGGAGATC-3′ (nt 306–328). *Dat* (Genebank accession number NM_010020.3): Forward: 5′- ACCTGGGCCCTCCACGGTGG-3′ (nt 849–868), Reverse: 5′- AAGGCAATCAGCACCCCAAA -3′ (nt 1149–1130). *Nestin* (Genebank accession number NM_016701.3): Forward: 5′- CAGGTCTCTCTTGGCTTTCCTG-3′ (nt 1074–1095), Reverse: 5′- GGTGAGGGTTGAGGGGTGG -3′ (nt 1512–1494). *Msx1* (Genebank accession number NM_010835.2): Forward: 5′- TGCTGCTATGACTTCTTTGCC -3′ (nt 264–284), Reverse: 5′- GCTTCCTGTGATCGGCCAT-3′ (nt 469–451). *βIII tubulin* (Genebank accession number NM_023279.2): Forward: 5′- ACCTTGTGTCTGCCACCATGA-3′ (nt 722–742), Reverse: 5′- TTACTCTGGATGGCCAGCAT 3′ (nt 1049–1030). *Pitx3* (Genebank accession number NM_008852.4): Forward: 5′- ACGCACTAGACCTCCCTCCAT-3′ (nt 119–139), Reverse: 5′- TACCAGTAGCCCGGGTACA-3′ (nt 595–577). *Vmat2* (Genebank accession number NM_172523.3): Forward: 5′- TTGCTCATCTGTGGCTGGG-3′ (nt 753–771), Reverse: 5′- TGGCGTTACCCCTCTCTTCAT-3′ (nt 823–803). *Ngn2* (Genebank accession number NM_009718.2): Forward: 5′- GACATTCCCGGACACACACC-3′ (nt 254–273), Reverse: 5′- CTCCTCGTCCTCCTCCTCGT-3′ (nt 446–427). For detection of cDNAs, the following protocol was used: denaturation at 95 °C for 5 min, the optimum number of cycles—depending on the primer pair—of PCR amplification were performed in the following conditions: denaturation at 95 °C for 45 s, annealing at an appropriate temperature for 45 s and elongation at 72 °C for 45 s. Final extension at 72 °C for 10 min was terminated by rapid cooling at 10 °C. Expression of genes of interest was normalized to *Gapdh* expression and quantified densitometrically. Differences between controls and experimental groups were tested for significance using the two-tailed unpaired Student’s *t* test. Results with levels of *p < 0.05, **p < 0.01 and ***p < 0.001 were considered significant.

### Immunoblotting

Cell monolayers from control and treated MN9D cells were washed with homogenization buffer containing (in mM) 280 mannitol, 10 HEPES, 10 KCl, 1 MgCl_2_, adjusted to pH 7.0 and a protease inhibitor “cocktail” (10 μM leupeptin, 2 mM benzamidine and 0.1 mM Pefabloc^®^SC), scraped off the culture flasks with a rubber policeman, pelleted by centrifugation at 250*g* for 5 min and resuspended in homogenization buffer. Homogenization was performed by sonication. Protein concentration was determined by Thermo Scientific NanoDrop 2000 Spectrophotometer and samples were processed for immunoblotting. Electrophoresis and blotting procedures were performed as previously described (Brandes et al. 2007). Blots were incubated with primary antibody overnight at dilutions 1:1000 for Gli1, Gli2, Gli3 and Ptch1 and 1:2000 and 1:10,000 for GAPDH. After incubation with secondary antibodies, blots were developed in enhanced chemiluminescence reagents and signals were visualized on X-ray film. Subsequently, films were scanned using a flat-bed scanner and the signal ratio antibody of interest, GAPDH, was quantified densitometrically. Differences in signal ratio between controls, BA- and SHH-treated undifferentiated and differentiated cells were tested for significance using two-tailed unpaired Student’s *t* test. Results with levels of *p < 0.05 and **p < 0.01 were considered significant.

## Results

**Fig. 1:**
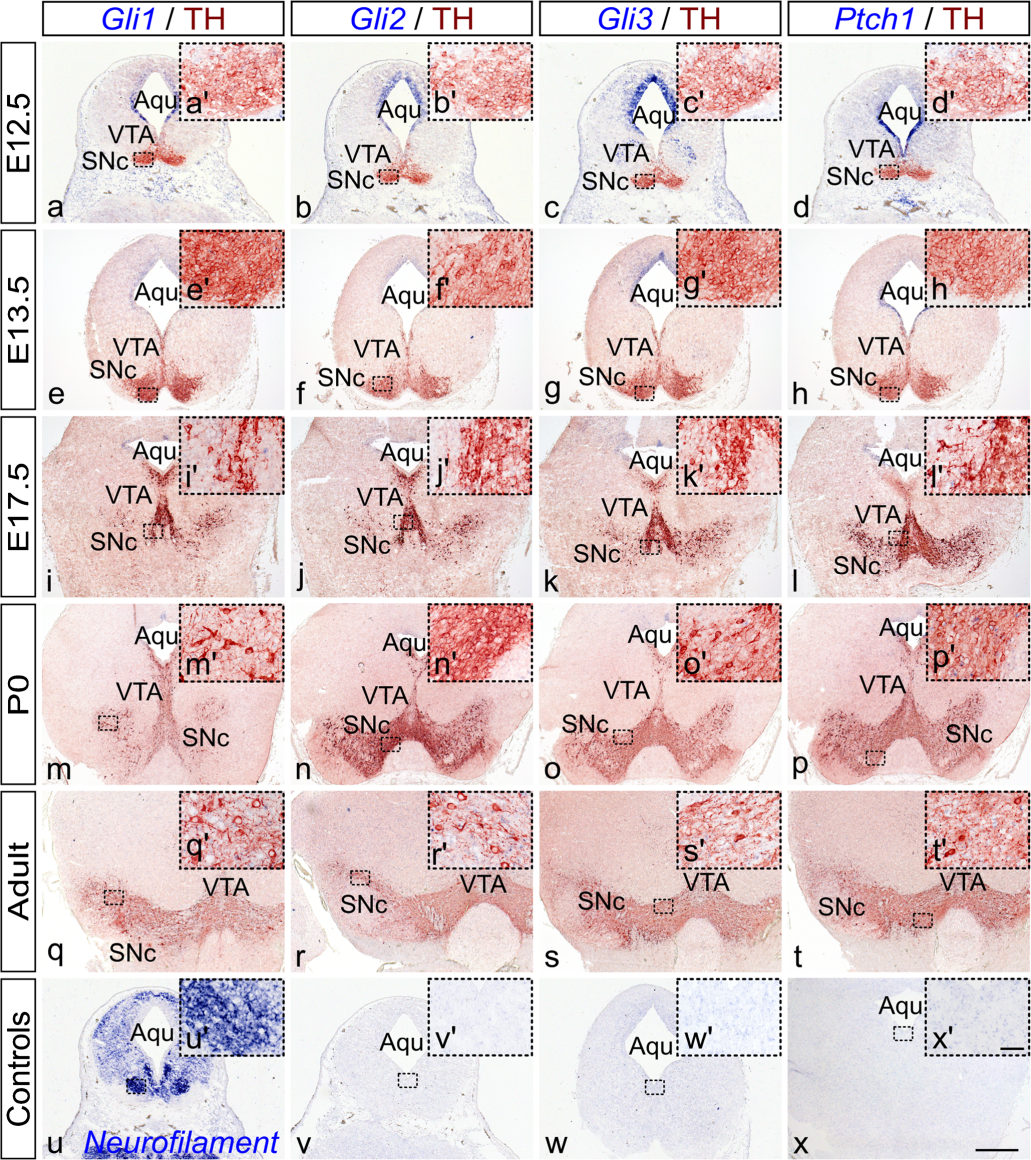
Expression pattern of SHH signaling components in mouse midbrain during embryonic development, in newborn and adult mice. RNA in situ hybridization with *Gli1* (*a, e, i, m, q*), *Gli2* (*b, f, j, n, r*), *Gli3* (*c, g, k, o, s*) and *Ptch1* (*d, h, l, p, t*) antisense probes. The analysis was performed on coronal fixed cryosections at embryonic day (E) (*E12.5*–*E17.5*), in sections from newborn (*P0*) and from 2 to 3 months old (*adult*) mice. TH immunolabeling was performed on the same sections, as highlighted in *insets* of the corresponding images. Expression domains of *Gli1* (*a*) do not overlap with TH (*a´*) at E12.5 mouse midbrain. At E12.5 and E13.5, *Gli1* and *Ptch1* were diffusely expressed both dorsally and ventrally and at the rostro-caudal axis and their expression became progressively restricted to the middle lateral domain of the mesencephalon. *Ptch1* expression (*d*) at the ventrolateral midbrain is complementary to that of *Gli2* (*b*) and *Gli3* (*c*), whose expression is also expanded dorsally. At E17.5 (*i–l*), P0 (*m–p*) and adult (*q–t*), expression of all examined genes was not co-localized with TH (*insets* *i´–* *t'*). Neurofilament expression was used as positive control for ISH labeling (*u*). Sense probes (*v–x*) were used to validate the specificity of the antisense probes and revealed no labeling.* Aqu* aqueduct,* SNc* substantia nigra pars compacta,* VTA* ventral tegmental area,* V3* third ventricle.* Scale bars* *a–x* 500 μm, *a´–x´*50 μm

To elucidate the spatial and temporal expression of crucial SHH signaling components during mouse midbrain embryonic development, we first determined the expression of the SHH receptor *Ptch1*, as well as of the transcription factors *Gli1*, *Gli2* and *Gli3* and established downstream determinants of the SHH signaling in mouse embryos at E12.5, E13.5 and E17.5 by in situ hybridization. Moreover, we used cryosections from newborn (P0) and adult (2–3 months old) mice to investigate expression of these SHH-signaling-associated genes at early postnatal stages and in adulthood. In order to be able to examine whether *Gli1*, *Gli2*, *Gli3* and *Ptch1* are expressed in mDA neurons, we performed immunolabeling for TH on the same sections (Fig. 1).

As shown in Fig. 1a, at E12.5, *Gli1* expression was detected and was restricted to the middle lateral and ventrolateral domains of the mesencephalon, while the area of substantia nigra pars compacta (SNC) and ventral tegmental area (VTA) neurons, as determined by TH immunolabeling, were devoid of *Gli1* expression (inset a´). *Gli2* (Fig. 1b) and revealed a comparable expression pattern with *Gli1*. There was some overlap between *Gli3* (Fig. 1c) and *Gli1* and *Gli2* expression at their domain boundaries, where *Gli* genes were expressed in a mosaic manner. *Ptch1* expression (Fig. 1d) was also restricted to the middle lateral domain of the mesencephalon. Taken together, at E12.5, expression domains of SHH-associated genes and TH (insets a´-d´) appeared to be clearly separated. At E13.5, expression of Gli family members did not obviously change, compared to that observed at E12.5. *Gli1* was predominantly expressed in the lateral mesencephalic domains (Fig. 1e), whereas *Gli2* (Fig. 1f) and *Gli3* (Fig. 1g) exhibited the same expression pattern comprising the dorsolateral parts of the midbrain that was complemented by *Ptch1* expression (Fig. 1h). *Ptch1* was found expressed in the ventrolateral domains of the midbrain (Fig. 1h). Again, similarly to E12.5, expression domains for *Gli1*, *Gli2*, *Gli3 and Ptch1* showed no overlapping with the area of mesencephalic TH-expressing neurons at all embryonic stages examined (insets e´–l´). The expression pattern was dampened after E12.5 and largely persisted in newborn (Fig. 1m–p and insets m´–p´) and adult (Fig. 1q–t and insets q´–t´) mice. Only at P0, expression of *Gli3* and *Ptch1* could be detected in the mDA neuronal area. For all ISH experiments, expression of the neuronal marker *neurofilament* was used as a positive control, as depicted in Fig. 1u (E12.5). To test the specificity of the probes, the corresponding sense probes were used and showed no labeling, as illustrated for *Gli2* at E12.5 (Fig. 1v), *Gli3* at E13.5 (Fig. 1w) and *Ptch1* at P0 (Fig. 1x).

**Fig. 2:**
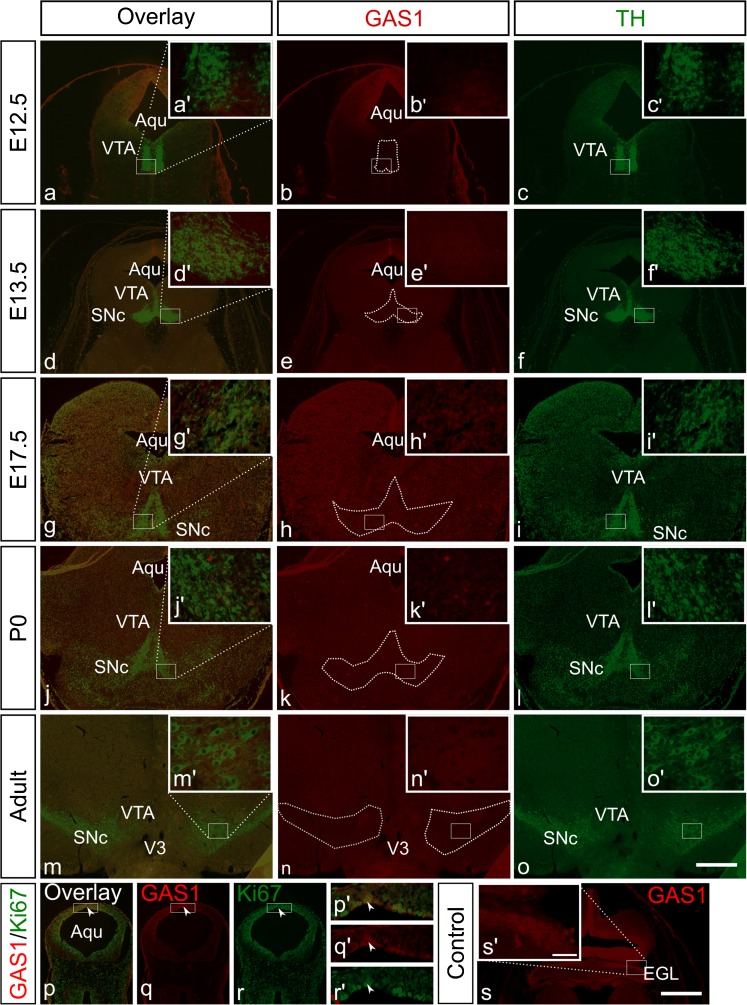
*a–o* GAS1 is not expressed in mouse midbrain dopaminergic neurons. Double immunofluorescence for GAS1 (*red*) and TH (*green*), on mouse midbrain coronal fixed tissue sections at embryonic day 12.5 (E12.5), E13.5, E17.5, newborn (P0) and adult mice. GAS 1 was expressed at dorsal mesencephalic domains at E12.5 but was not expressed at the area of TH positive neurons at any stage investigated.* Insets* *a´–o´* represent higher magnification of the* white boxed area* of the respective images. The area outlined by the* dotted line* corresponds to the area of midbrain TH-expressing cells. *p–r* double immunofluorescence for GAS1 (*red*) and the cell proliferation marker Ki67 (*green*) at E11.5 reveals co-localisation of the proteins (*arrowheads*).* Insets* *p´–r´* represent higher magnification of the* white boxed area* of the respective images. *s* Immunofluorescence for GAS1 in frontal sections of the developing cerebellum, a tissue used as positive control.* Aqu* aqueduct,* EGL* external germinal layer;* SNc* substantia nigra pars compacta,* VTA* ventral tegmental area;* V3* third ventricle.* Scale bar* for all images, 500 μm; for all insets, 50 μm

Since none of the examined players of SHH signaling were expressed in the area where dopaminergic neurons are located, we next examined the abundance of GAS1, identified to function as an accessory SHH receptor that may modulate SHH signaling (Tenzen et al. 2006). To ensure putative co-localization of GAS1 in TH-expressing dopaminergic neurons (area outlined by dotted lines in Fig. 2b, e, h, k, and n), we performed double immunofluorescence for GAS1 and TH during embryonic development, i.e., E12.5 (Fig. 2a–c), E13.5 (Fig. 2d–f) and E17.5 (Fig. 2g–i), in P0 (Fig. 2j–l) and in adult mice (Fig. 2m–o). As shown in Fig. 2, GAS1 immunolabeling was only detectable at E12.5 and was restricted to the dorsal mesencephalic domains (Fig. 2b). The ventral midbrain area containing TH positive neurons at E12.5 (high-magnification insets) was devoid of GAS1 immunoreactivity. From E13.5 onwards protein abundance of GAS1 was no longer detectable in the midbrain, thus again, demonstrating lack of expression in the area of differentiated TH-expressing neurons. This absent labeling pattern for GAS1 persisted in the midbrain of newborn (Fig. 2j–l) and of adult (Fig. 2m–o) mice. In all cases, the area of mDA neurons (higher magnification insets in the corresponding images) was devoid of GAS1 immunoreactivity. To ensure specificity GAS1 labeling, fixed sections from the developing mouse cerebellum were used as a positive control. As shown in Fig. 2s, GAS1 immunofluorescence could be observed in the external germinal layer (EGL) of the cerebellum. Moreover, to examine whether GAS1 is preferentially expressed in areas where proliferation takes places, double immunofluorescence with GAS1 and Ki67 was performed at E11.5 (Fig. 2p–r). Indeed, higher magnification images (Fig. 2p´–r´) show co-localization (arrowheads) of the proteins in the majority of cells.

**Fig. 3:**
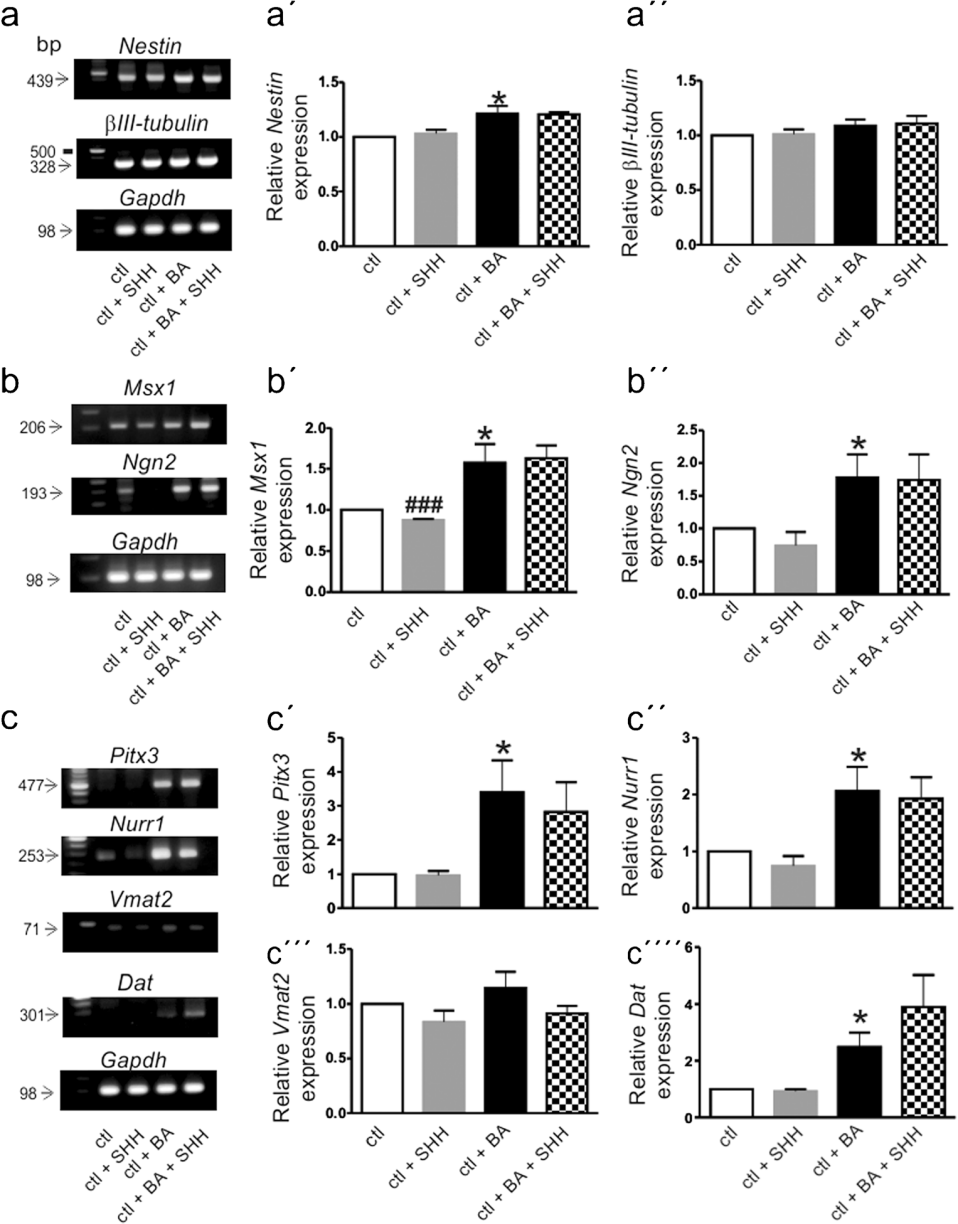
Expression and regulation of the stem cell marker *Nestin* (*a*, a´) and neuronal marker *βIII-tubulin* (*a, a´´*), the dopaminergic neuronal progenitor markers *Msx1* (*b, *b´) and *Ngn2* (b, b´´) and the midbrain dopaminergic neuronal markers *Pitx3* (c, c´), *Nurr1* (*c, c´´*), *Vmat2* (*c, c´´´*) and *Dat* (*c, c´´´´*) in undifferentiated (*Ctl*) or differentiated (*ctl + BA*) MN9D cells in the presence (*+SHH*) or absence of exogenous SHH by RT-PCR. Cultured MN9D cells were treated with 1 mM butyric acid (*BA*) for at least 6 days and/or with 1 nM SHH for 48 h, followed by RT-PCR analysis. Transcript expression of the examined genes was normalized to *Gapdh*. *p < 0.05, indicates statistically significant increase relative to control and ^###^ *p* < 0.001 indicates statistically significant decrease relative to control as assessed by two-tailed unpaired Student’s *t* test (*n* = 3). Data are given as fold changes compared to control and *error bars* represent SEM from three independent cultures and experiments

We next sought to investigate the expression pattern of the stem cell marker *Nestin,* lineage markers *(β-III tubulin* and *Gfap*, for neuronal and glial lineage, respectively), of major dopaminergic neuronal progenitor markers (*Msx1, Ngn2*), midbrain dopaminergic neuronal markers (*Nurr1, Pitx3, Dat, Vmat2*), and markers of SHH signaling pathway (*Ptch1*, *Gli1*, *Gli2* and *Shh*) in undifferentiated and differentiated (treated with 1 mM butyric acid for at least 6 days) MN9D cells. MN9D is an established cell line to study mechanisms of differentiation and survival of midbrain dopaminergic neurons (Hermanson et al. 2003). Undifferentiated and differentiated cells were treated with 1 nM SHH for 48 h and subsequently expression of the aforementioned genes was examined by RT-PCR. Transcript expression was normalized to *Gapdh* expression. As shown in Fig. 3, expression of the stem cell marker *Nestin* (Fig. 3a, a´; 439 bp) was observed in control MN9D cells and treatment with BA significantly increased transcript expression, compared to the untreated controls (1.21 ± 0.07-fold; *p < 0.05, using two-tailed unpaired Student’s *t* test, *n* = 3). Transcript expression for *βIII-tubulin* was also detectable in control (Fig. 3a, a´´; 328 bp; ctl; undifferentiated and untreated cells) MN9D cells but neither treatment with butyric acid (ctl + BA; 1.09 ± 0.05-fold) nor with SHH (ctl + SHH: 1.02 ± 0.04-fold and ctl + BA +SHH: 1.10 ± 0.07-fold) changed expression levels (not significant, using the two-tailed unpaired Student’s *t* test, *n* = 3). Expression of the glial marker *Gfap* could not be detected in any experimental group (data not shown).

Figure 3b illustrates expression and regulation of dopaminergic neuronal progenitor markers *Msx1* (Fig. 3b, b´) and *Ngn2* (Fig. 3b, b´´) in undifferentiated and differentiated MN9D cells with or without SHH treatment. Transcript expression was normalized to *Gapdh* expression. In undifferentiated MN9D cells, following exposure to SHH expression of *Msx1* (Fig. 3b, b´; 206 bp), it was significantly reduced (0.87 ± 0.01-fold, ^###^ *p* < 0.001 for significant decrease using the two-tailed unpaired Student’s *t* test, *n* = 3) but was significantly increased after treatment with butyric acid (1.57 ± 0.22-fold; *p < 0.05 for significant increase using the two-tailed unpaired Student’s *t* test, *n* = 3). With regard to *Ngn2* expression (Fig. 3b, b´´; 193 bp), differentiation of MN9D cells with 1 mM butyric acid, significantly upregulated *Ngn2* expression (1.78 ± 0.35-fold; *p < 0.05 for significant increase using the two-tailed unpaired Student’s *t* test, *n* = 3), compared to undifferentiated cells.

Gene expression of specific midbrain dopaminergic neuronal markers (Fig. 3c) was considerably changed after treatment of MN9D cells with 1 mM butyric acid. Again, transcript expression was normalized to *Gapdh* expression. As shown in Fig. 3c, *Pitx3* expression (Fig. 3c, c´; 477 bp), *Nurr1* (Fig. 3c, c´´; 253 bp) and *Dat* (Fig. 3c, c´´´´; 301 bp) expression were significantly increased in butyric acid treated cells (3.39 ± 0.94-fold, 2.06 ± 0.42-fold and 2.48 ± 0.50-fold for *Pitx3, Nurr1* and *Dat*, respectively, *p < 0.05, using the two-tailed unpaired Student’s *t* test, *n* = 3), compared to undifferentiated control and SHH-treated (0.97 ± 0.12-fold; 0.74 ± 0.16-fold) cells. However, treatment of differentiated MN9D cells with SHH was not able to significantly potentiate the effects of butyric acid alone (2.82 ± 0.87-fold, 1.92 ± 0.37-fold and 3.89 ± 1.11-fold for *Pitx3*, *Nurr1* and *Dat*, respectively). In contrast, expression of *Vmat2* (Fig. 3c, c´´´; 71 bp) was comparable in all experimental groups (0.83 ± 0.10-fold, 1.14 ± 0.14-fold and 0.91 ± 0.07 for undifferentiated treated with SHH, differentiated and differentiated and treated with SHH, respectively; not significant, *n* = 3).

**Fig. 4:**
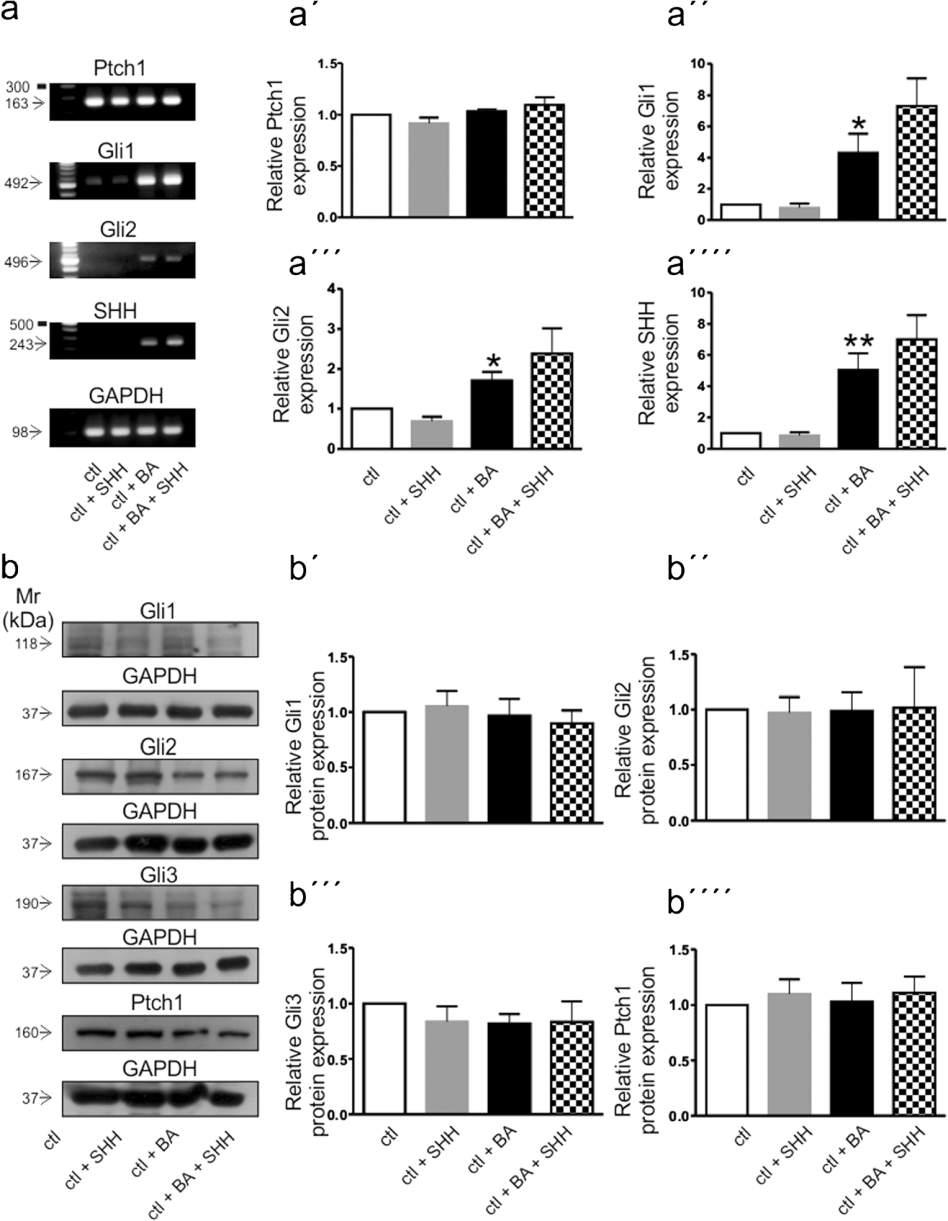
**a** Expression and regulation of the SHH signaling markers *Ptch1* (*a, a´*), *Gli1* (*a, a´´*), *Gli2* (*a, a´´´*) and *Shh* (*a, a´´´´*) in undifferentiated (*Ctl*) or differentiated (*ctl + BA*) MN9D cells in the presence (*+SHH*) or absence of exogenous SHH by RT-PCR. Cultured MN9D cells were treated with 1 mM butyric acid (*BA*) for at least 6 days and/or with 1 nM SHH for 48 h, followed by RT-PCR analysis. Transcript expression of the examined genes was normalized to *Gapdh*. *p < 0.05 and **p < 0.01 indicate statistically significant differences relative to control, as assessed by two-tailed unpaired Student’s *t* test (*n* = 3). Data are given as fold changes compared to control and *error bars* represent SEM from three independent cultures and experiments. **b** Immunoblotting for GLI1 (*b, b´*), GLI2 (*b, b´´*), GLI3 (*b, b´´´*) and PTCH1 (*b, b´´´´*) in MN9D cells and following treatment with 1 mM butyric acid (BA) in the presence or absence of 1 nM SHH. Not significant after densitometric analysis of the respective protein, GAPDH and two-tailed unpaired Student’s *t* test, *n* = 3, compared to the untreated controls; 30 μg protein was loaded per lane. Representative for three independent cultures and experiments

The expression pattern of genes associated with SHH signaling and their putative regulation through differentiation and/or treatment with SHH is shown in Fig. 4. Transcripts for *Ptch1* (Fig. 4a; 163 bp), *Gli1* (Fig. 4a; 492 bp), *Gli2* (Fig. 4a; 496 bp) and *Shh* (Fig. 4a; 243 bp) were detectable in control undifferentiated MN9D cells. Treatment of undifferentiated cells with SHH did not change expression levels of the genes, whereas differentiation with butyric acid, again, significantly increased expression of *Gli1* (Fig. 4a´´; 4.31 ± 1.21-fold), *Shh* (Fig. 4a´´´´; 5.05 ± 1.05-fold) and *Gli2* (Fig. 4a´´´; 1.71 ± 0.21-fold), compared to the undifferentiated untreated controls. SHH treatment of differentiated MN9D cells caused significant upregulation of the genes, (7.31 ± 1.75-fold, 7.01 ± 1.55-fold and 2.37 ± 0.63-fold, for *Gli1*, *Shh* and *Gli2*, respectively) compared to the undifferentiated and SHH-treated cells (*p < 0.05 and **p < 0.01 using the two-tailed unpaired Student’s *t* test, *n* = 3) but not significant compared to cells treated with butyric acid alone. In contrast, *Ptch1* expression was comparable in all experimental groups (Fig. 4a´; 0.91 ± 0.05-fold, 1.03 ± 0.01-fold and 1.09 ± 0.07-fold for undifferentiated treated with SHH, differentiated and differentiated and treated with SHH, respectively).

As a next step, we validated the PCR data at the protein level. The results are shown in Fig. 4b. Using antibodies against GLI1, GLI2, GLI3 and PTCH1, immunoreactive bands at ∼118, ∼167, ∼190 and ∼160 kDa were detected in undifferentiated MN9D cells, corresponding to the full length of the respective proteins. Protein levels were normalized to GAPDH. However, in contrast to the PCR data, expression levels of the proteins were comparable between undifferentiated and butyric acid-treated MN9D cells (0.96 ± 0.15-fold, 0.98 ± 0.16-fold, 0.82 ± 0.08-fold and 1.03 ± 0.17-fold for GLI1 (Fig. 4b´), GLI2 (Fig. 4b´´), GLI3 (Fig. 4b´´´) and PTCH1 (Fig. 4b´´´´), respectively), as well as between SHH-treated (0.89 ± 0.11-fold, 1.01 ± 0.36-fold, 0.83 ± 0.18-fold and 1.10 ± 0.14-fold for GLI1, GLI2, GLI3 and PTCH1, respectively) and untreated cells (not significant, using the two-tailed unpaired Student’s *t* test, *n* = 3).

**Fig. 5:**
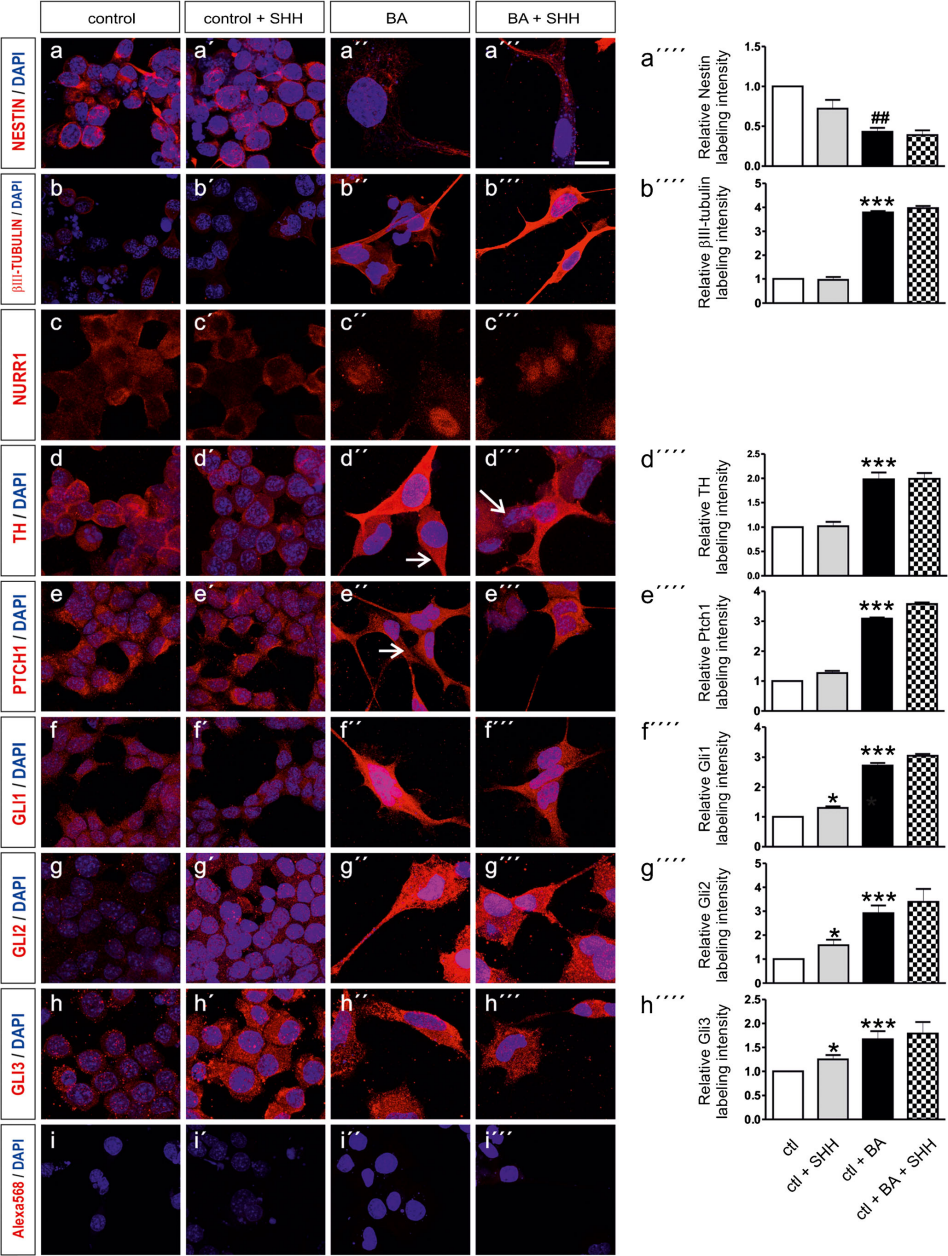
Cultured MN9D cells were treated for at least 6 days with 1 mM butyric acid (*BA*) and for 48 h with 1 nM SHH (*+SHH*), followed by immunolabeling for NESTIN (*a–a´´´*), βIII-TUBULIN (*b–b´´´*), NURR1 (*c–c´´´*), TH (*d–d´´´*), PTCH1 (*e–e´´´*), GLI1 (*f–f´´´*), GLI2 (*g–g´´´*) and GLI3 (*h–h´´´*). Nuclei are labeled with DAPI. Undifferentiated MN9D cells were immunopositive for all proteins investigated at a low level. In differentiated, i.e., BA-treated cells, a neuronal morphology is evident and labeling intensity of most of the examined proteins is considerably increased. Only NESTIN immunoreactivity was decreased after BA treatment.* Arrows* indicate cells that remained undifferentiated despite BA treatment. (*i–i´´´*) Omission of primary antibody revealed no labeling. *a´´´´–h´´´´* Quantification of labeling intensity normalized to the untreated controls. *p < 0.05 and ***p < 0.001 indicate statistically significant increase relative to control, ^##^ *p* < 0.05 indicates statistically significant decrease, as assessed by two-tailed unpaired Student’s *t* test (*n* = 3). Data are given as fold changes compared to control and *error bars* represent SEM from three independent cultures and experiments. *Scale bar* 25 μm

Figure 5 illustrates immunolocalisation of NESTIN (Fig. 5a–a´´´), βIII-TUBULIN (Fig. 5b–b´´´), NURR1 (Fig. 5c–c´´´), TH (Fig. 5d–d´´´), PTCH1 (Fig. 5e–e´´´), GLI1 (Fig. 5f–f´´´), GLI2 (Fig. 5g–g´´´) and GLI3 (Fig. 5h–h´´´) by immunofluorescence and confocal microscopy in undifferentiated (5a–5 h), and differentiated (Fig. 5a´´–h´´), i.e., BA-treated MN9D cells in the presence (Fig. 5a´–h´ and a´´´–h´´´) or absence of 1 nM exogenous SHH. All examined proteins were, though at low level, present in undifferentiated and untreated MN9D cells, exhibiting diffuse intracellular distribution. After treatment with BA, many cells adopted a neuronal phenotype, however, as shown in Fig. 5 (arrows), many MN9D cells remained undifferentiated and revealed morphology and labeling intensity comparable to the controls. Quantification of the labeling (Fig. 5a´´´´–h´´´´) showed that treatment of undifferentiated cells with SHH significantly increased labeling intensity for GLI1, GLI2 and GLI3 (1.30 ± 0.05-fold, 1.58 ± 0.23-fold and 1.25 ± 0.09 for GLI1, GLI2 and GLI3, respectively, *p < 0.05, using the two tailed unpaired Student’s *t* test, *n* = 3), compared to untreated controls. In contrast, labeling intensity of NESTIN, β-III-TUBULIN, TH, and PTCH1 was comparable between controls in the absence or presence of exogenous SHH (Fig. 5a´´´´–h´´´´, 0.72 ± 0.11-fold, 0.96 ± 0.12-fold, 1.02 ± 0.09-fold and 1.27 ± 0.07-fold, for Nestin, β-III tubulin, TH and Ptch1, respectively). Differentiation of MN9D cells with 1 mM butyric acid, as demonstrated in cells that have acquired a neuronal morphology, significantly decreased labeling intensity of Nestin (Fig. 5a´´´´; 0.43 ± 0.05; ^##^ *p* < 0.01, using the two tailed unpaired Student’s *t* test, *n* = 3), compared to undifferentiated cells and significantly increased intensity of βIII-TUBULIN (Fig. 5b´´´´; 3.79 ± 0.06-fold), TH (Fig. 5d´´´´; 1.98 ± 0.14-fold), PTCH1 (Fig. 5e´´´´; 3.09 ± 0.04-fold), GLI1 (Fig. 5f´´´´; 2.72 ± 0.09-fold), GLI2 (Fig. 5g´´´´; 2.92 ± 0.32-fold) and GLI3 (Fig. 5h´´´´; 1.67 ± 0.17-fold) (***p < 0.001, using the two-tailed unpaired Student’s *t* test, *n* = 3). With regard to NURR1 (Fig. 5c–c´´´), translocation of immunolabeling to the nucleus was observed following treatment of the cells with BA in the presence or absence of exogenous SHH. Treatment of the cells with BA together with SHH significantly increased labeling intensity of all but for Nestin proteins compared to the controls, but did not further increase the labeling intensity observed by treatment with BA alone (Fig. 5a´´´´–h´´´´, 0.39 ± 0.06-fold, 3.97 ± 0.09-fold, 1.99 ± 0,12-fold, 3.58 ± 0.05-fold, 3.05 ± 0.06-fold, 3.39 ± 0.54-fold and 1.79 ± 0.24-fold for Nestin, β-III tubulin, TH, Ptch1, Gli1, Gli2 and Gli3, respectively). No labeling could be observed when cells were incubated only with secondary antibody (Fig. 5i–i´´´).

Taken together, these results suggest that expression and distribution of SHH signaling components considerably differ between MN9D cells and native mouse mesencephalic tissue.

## Discussion

In the present study, we sought to investigate expression of key molecules associated with SHH signaling during mouse mDA neuron development at embryonic day 12.5 (E12.5) onwards, as well as in midbrain of newborn (P0) and of adult (2–3 months old) mice. Using in situ hybridization and immunohistochemistry on the same tissue sections with specific RNA probes and antibodies against TH, respectively, we show that expression of all genes studied, namely *Ptch1*, *Gli1*, *Gli2* and *Gli3,* do not overlap with TH at any embryonic or postnatal stages investigated, thus demonstrating that these genes are not expressed in the area where midbrain dopaminergic neurons are located (Fig. 1). At embryonic stages, expression of *Ptch1*, *Gli1*, *Gli2* and *Gli3* revealed a mosaic expression pattern between the mesencephalic domains, with some overlap between *Gli1* and *Ptch1* expression, being confined ventrolaterally and *Gli2* and Gli3 expression being restricted dorsolaterally at their domain boundaries (Fig. 1).

The temporal dynamic expression of SHH during mDA neuron development is one of the key events in establishing mDA neuron diversity (Joksimovic et al. 2009a; Blaess et al. 2011). SHH is secreted by the notochord and the floor plate and modulates the expression of FoxA2 via Gli1 between E8 and E8.5, thereby playing a key role in the induction of a dopaminergic phenotype (Bayly et al. 2012). In response to SHH signaling, *Gli1* expression is upregulated by *Gli2*, whereas *Gli3* is suppressed by SHH (Blaess et al. 2006). SHH also initiates the expression of *Lmx1a*, which in turn induces the expression of *Msx1* (Andersson et al. 2006). The question whether SHH is critically involved in the differentiation of mDA neurons has been controversially discussed in the literature. The early studies by Ye et al. (1998), Hynes et al. (1995, 1997) and Hynes and Rosenthal (1999) supported the notion that SHH is necessary and sufficient for mDA neuron differentiation. Our previous studies in vitro also showed that SHH is required for induction and survival of mDA neurons. Treatment of primary dissociated mDA neurons from rat E12 or E14 with exogenous SHH significantly increased the number of TH-positive cells, whereas neutralization of endogenous SHH significantly reduced the number of TH-positive cells (Farkas et al. 2003; Roussa et al. 2004). Importantly, these SHH effects could only be observed in the absence of endogenous TGF-β, indicating that SHH cooperates with other signaling molecules to exert its actions. SHH signaling has even also been shown to be required for the normal development of both dorsal and ventral domains of the anterior midbrain (Ishibashi and McMahon 2002). In contrast, more recent studies have challenged the role of SHH during the development of mDA neurons. SHH inhibits progenitors to acquire a dopaminergic cell fate (Joksimovic and Awatramani 2014; Joksimovic et al. 2009b; Tang et al. 2010). Other studies have proposed the scenario that SHH is crucial for the development of mDA neurons exclusively during the time window E8.5–E11, acting as an early patterning molecule of the floor plate and midbrain area (Mesman et al. 2014; Blaess et al. 2006), thus supporting the view that SHH is only indirectly involved in the differentiation of mDA neurons. In line with this notion, the smoothened receptor function is apparently not required for the maturation and survival of mDA neurons during late development, aging or under stress challenge (Zhou et al. 2016). In the study of Hayes et al. (2011), the authors investigated the contribution of the SHH-secreting versus the SHH-responding cells to the mDA neuron domains and proposed that the developmental order from the anterior to the posterior domain is established within the progenitors before they terminally differentiate and that the timing of gene expression of multiple lineages, including SHH and Gli1, is the underlying event for mDA neuron heterogeneity. Our results at E12.5 are consistent with previous observations at E11.5 (Blaess et al. 2011; Tang et al. 2010). Since SHH and SHH-related molecules have been shown to reveal dynamic temporal–spatial changes, we extended the analysis to later embryonic stages, newborn and adult neurons and show that the expression pattern observed at E12.5 persisted until E13.5, whereas at E17.5, P0 and adult mice expression was faint or absent in the mesencephalic domain. The previously observed in vitro responsiveness of differentiated TH-expressing neurons to SHH may therefore be considered as a result of early patterning events. It can also be hypothesized that an alternative SHH receptor might mediate SHH effects.

In addition to Ptch1, other receptors like Cdon, Boc and Gas1 are identified as an accessory and potent to positively modulate SHH signaling activity. Gas1, Cdo and Boc play essential and overlapping roles during SHH-mediated patterning of the neural tube (Allen et al. 2011). Gas1, Cdo and Boc are required and cooperate in the promotion of SHH signaling during embryonic development (Cole and Krauss 2003). Whereas Boc and Gas1 each form distinct SHH receptor complexes with Ptch1 and are required for SHH-mediated cell proliferation, Cdon and Boc promote SHH-dependent cell fate specification. Izzi et al. (2011) provided evidence for the biological relevance and requirement of Boc and Gas1 in Hedgehog (Hh) signaling. A mutated Hh ligand that binds Ptch1 but not Boc, Cdon, or Gas1 cannot activate Hh signaling in cerebellar granule neuron progenitors. We asked whether Gas1 protein is expressed in the developing, newborn and adult midbrain and whether its expression overlaps with that of TH. Therefore, we performed double immunofluorescence for Gas1 and TH on mouse tissue sections (Fig. 2). Gas1 protein could only be detected in the midbrain at E11.5 and E12.5, its distribution being restricted to the dorsal mesencephalic domain where it co-localizes with Ki67 (Fig. 2p–r and p´–2r´). These results extend previous studies in which Gas1 was found to be involved in the development of the nervous system as being initially expressed in ventral progenitors of the spinal cord and in germinal niches of developing dentate gyrus and cortex (Estudillo et al. 2016). However, at the time points investigated, Gas1 expression could not be detected in the proliferating area of the midbrain floor plate, where progenitors of mDA neurons are located. These data suggest that, although Gas1 maybe preferentially expressed at time points where proliferation takes place and/or in proliferative active cells, it has no impact on the proliferation of mDA progenitors at E11.5 and E12.

MN9D is a cell line extensively used to study differentiation processes of mDA neurons. Since MN9D cells express TH, are capable of dopamine transport and release, express voltage-activated Na^+^ channels and are sensitive to MPP^+^, they have been appreciated as a suitable model to investigate mDA neurons (Choi et al. 1991; Chen et al. 2005). MN9D cells can be differentiated following exposure to 1 mM butyric acid and hence they acquire electrophysiological properties resembling substantia nigra pars compacta neurons (Rick et al. 2006). In addition, differentiated MN9D cells produce an increased quantal size compared with undifferentiated MN9D cells (Dong et al. 2008). We asked whether expression of the genes of interest in undifferentiated MN9D cells, as well as upon differentiation with butyric acid, corresponds to the in vivo situation (Figs. 3, 4, 5). Moreover, we investigated whether this cell line is responsive to exogenous SHH and whether exposure of MN9D cells to SHH might influence expression and distribution of SHH-signaling-associated genes. To that end, we first monitored butyric acid-induced differentiation in MN9D cells in the presence and absence of exogenous SHH by assessing gene expression and protein abundance of *Nestin*, as a representative molecular marker for stem cells, of *βIII-tubulin* and *Gfap* as key markers for the neuronal and glial lineage, respectively, of *Msx1* (Vallstedt et al. 2001) and *Ngn2* (Bertrand et al. 2002), as exemplary for dopaminergic neuronal progenitor markers and *Pitx3* (Smidt et al. 2004), *Nurr1* (Wallén and Perlmann 2003), dopamine transporter *Dat* and *Vmat2* as specific markers for midbrain dopaminergic neuronal markers.

With only one exception, namely *Msx1*, exposure of undifferentiated MN9D cells to SHH caused no effect on the transcript expression of any of the investigated genes. In contrast, treatment of MN9D cells with butyric acid for at least 6 days significantly upregulated expression of *Msx1*, *Ngn2* and of the late dopaminergic markers *Pitx3*, *Nurr1*, *Vmat2* and *Dat*, thus being consistent with the notion of differentiation towards the midbrain dopaminergic lineage. With regard to the questions addressed in the present work, it was surprising that the investigated SHH-signaling-related genes, *Ptch1, Gli1* and *Gli2*, were expressed at considerable levels in undifferentiated MN9D cells but their expression was not influenced upon exposure to SHH. In contrast, differentiation of MN9D cells was associated with robust upregulation of *Gli1* and *Gli2* but not of *Ptch1*. Moreover, while expression of the ligand SHH was hardly detectable in undifferentiated MN9D cells, it was clearly detectable following treatment with butyric acid. Due to the high SEM values, the obvious tendency for increased expression in the investigated genes in the presence of exogenous SHH did not reach statistical significance. In BA-treated cells (without SHH treatment), SEM values were also increased, probably reflecting the heterogeneity of the cultures, as will be discussed below. In contrast to the PCR data, at the protein level, we could not find any differences in abundance of GLI1, GLI2, GLI3 or PTCH1 between undifferentiated and differentiated MN9D cells. One reason could be that the RNA and protein isolation were performed at the same time point. This fact, together with the consideration that, even after butyric acid treatment, cultured MN9D cells are inhomogeneous with regard to their differentiation state, i.e., consisting of cells that are still at the progenitor stage, while others have already acquired a neuronal phenotype, makes it reasonable to assume that the effect of butyric acid has likely been masked. This argument is supported by the results obtained by immunofluorescence (Fig. 5), where it is clearly demonstrated that, although the labeling intensity of TH, GLI1, PTCH1 and βIII-TUBULIN in MN9D cells exhibiting a neuronal morphology was dramatically increased following butyric acid treatment compared to undifferentiated MN9D cells, many cells remained undifferentiated (arrows) and revealed a labeling intensity comparable to the controls. When the labeling intensity of the proteins was quantified in differentiated cells only, i.e., in cells that have acquired a neuronal morphology, expression of Nestin was significantly increased, accompanied by significant decrease of β-III-tubulin expression. The immunocytochemical analysis also revealed that, in differentiated cells, immunolabeling for TH, PTCH1, GLI1, GLI2 and GLI3 was significantly increased. Taken together, the in vitro results suggest that the expression pattern of SHH signaling components in MN9D cells differs considerably from that of native mouse mecencephalic tissue. The MN9D cell line is a valuable model for investigating early development but not for the differentiation and survival of mDA neurons.
